# Evaluation of the safety and efficacy of a Fuling-Zexie decoction for people with asymptomatic hyperuricemia: protocol for a prospective, double-blinded, randomized, placebo-controlled clinical trial

**DOI:** 10.1186/s13063-022-06479-3

**Published:** 2022-06-20

**Authors:** Jingyao Yan, Yingyan Zhou, Qiaowen Yang, Jiaqi Wu, Xiaohong He

**Affiliations:** 1 Department of Rheumatology, Guangdong Provincial Hospital of Chinese Medicine, No. 55 of Neihuanxi Road, Higher Education Mega Center, Guangzhou, 510006 China; 2 State Key Laboratory of Dampness Syndrome of Chinese Medicine, The Second Affiliated Hospital of Guangzhou University of Chinese Medicine, No.111 Dade Road, Yuexiu District, Guangzhou, 510120 China

**Keywords:** Fuling-Zexie decoction, Asymptomatic hyperuricemia, Double-blinded, Randomized, Placebo-controlled

## Abstract

**Background:**

Hyperuricemia increases the risk of gout and cardiovascular complications, and how to manage asymptomatic hyperuricemia is controversial. Randomized controlled trials and comparative studies are needed to guide management and treatment. Studies show that Chinese medicine can decrease uric acid through multiple targets, but many of these studies have been conducted in animals because of the lack of a consistent prescription and mechanism. Therefore, we designed this research to study whether Chinese medicine is truly effective and which target is essential by using an approved prescription of a Fuling-Zexie decoction to further guide large sample experiments to determine whether Chinese medicine can reduce the long-term incidence of gout and cardiovascular events.

**Methods:**

This pilot study is a prospective, double-blinded, randomized, placebo-controlled clinical trial developed from March 2020 to December 2021. Thirty people with asymptomatic hyperuricemia will be recruited and assigned to either the Chinese medicine group or placebo group, and each group will have 15 subjects. During the 12-week observation period, there will be 4 visits. The decline in uric acid is the main outcome measure, and urinary uric acid, inflammatory biomarkers, and other indices that may be involved in lowering uric acid are the secondary outcome measures.

**Discussion:**

This study will probe the effect of Chinese medicine treatment on hyperuricemia and explore possible therapeutic mechanisms. By performing this trial, we hope to provide evidence and data to support further large clinical studies.

**Trial registration:**

ChiCTR2000038575. Registered on September 24, 2020.

**Supplementary Information:**

The online version contains supplementary material available at 10.1186/s13063-022-06479-3.

## Background

As living standards rise and dietary habits change, the prevalence of hyperuricemia has increased [1], especially in China [2]. It has become the second most common metabolic disease after diabetes mellitus and affects 133 persons per 1000 inhabitants in mainland China (the morbidity for men is 19.4%, and 7.9% for women) [3]. Meanwhile, the prevalence rate is higher in cities than in rural areas, and statistics show that the incidence of hyperuricemia in elderly individuals in Guangzhou and Beijing is 13.2% and 17.6%, respectively [4]. Hyperuricemia may eventually lead to gout and augment the risk of hypertension and other cardiovascular diseases [5]. However, there is no consensus on the management of asymptomatic hyperuricemia, and it remains controversial [6]. Chinese and Japanese guidelines recommend considering drug therapy when people have a serum urate level of no less than 9.0 mg/dL or 8.0 mg/dL with complications to prevent gout and cardiovascular or renal impairment [7, 8]. India’s Integrated Diabetes & Endocrine Academy (IDEA) has similar opinions [9], while asymptomatic hyperuricemia should only be treated with nonpharmacological treatment, which is the general consensus in the USA and Europe [10, 11]. The Asia-Pacific League of Associations for Rheumatology recommends against urate-lowering therapy for people with asymptomatic hyperuricemia and hypertension and gives no specific recommendations for people with asymptomatic hyperuricemia and chronic kidney disease due to insufficient evidence [12]. There are few studies on the treatment of asymptomatic hyperuricemia, including drug and nondrug treatments. These studies indicate benefits in treating people with asymptomatic hyperuricemia combined with insulin resistance or type 2 diabetes [13, 14] and an opposite result in mitigating the decline in kidney function [15, 16]. There is a need to conduct more research.

Influenced by the Chinese culture of “nipping problems in the bud,” people with hyperuricemia seek early interventions to prevent progression to gout and other diseases. Nonetheless, they do not prefer uric acid-lowering drugs, as someone with hyperuricemia is considered “not ill.” Thus, many people seek out Chinese medicine treatments. There have been randomized, double-blind, placebo-controlled trial on acupuncture for asymptomatic hyperuricemia [17], but no studies on Chinese medicine have been conducted.

Some animal studies have shown that Chinese medicine can lower uric acid levels, relieve symptoms and reduce the frequency of gout attacks [18–24]. The mechanism of Chinese medicine is complex and not yet clear. Some studies indicate that Chinese medicine may promote uric acid excretion [18–20]. Other studies have shown that Chinese medicine can modulate the levels of inflammatory cytokines [21–24]. Evidence also suggests that Chinese medicine inhibits the synthesis of uric acid [25, 26]. Therefore, our trial will test subjects’ uric acid excretion capacity and inflammation index. Recently, trials have found evidence for a difference in intestinal microecology between hyperuricemic people and healthy people [27, 28]. Experiments conducted in rats have shown that Chinese medicine has a certain influence on the composition of intestinal flora [29, 30]. Therefore, intestinal microecology before or after Chinese medicine treatment is another potential target of interest.

Evaluating the efficacy of Chinese medicine treatments and developing effective treatments for asymptomatic patients with hyperuricemia is difficult because Chinese medicine treats patients based on syndrome differentiation rather than disease differentiation; thus, the recipes for Chinese medicine for people may vary in composition. Therefore, it is essential to establish syndrome standards. Due to the lack of relevant literature and research, we gathered information on classic Chinese medicine by consulting Chinese medicine experts and combining their clinical experience to reach a consensus that for people with hyperuricemia in Guangdong Province, dampness syndrome is the main affliction.

We designed questionnaires that were completed by 11 people with hyperuricemia using an electronic questionnaire system. The results showed that all 11 cases were consistent with the clinical manifestations of dampness syndrome. Eight individuals had the manifestations of cold-dampness syndrome, and 3 showed the manifestations of dampness-hot syndrome. The minimum dampness score was 43, the maximum was 104, and the average was 70.09 ± 17.56. In summary of the 11 cases, 54.5% had a superficial syndrome. Based on clinical experience and combining the concept in Chinese medicine that “the beginning of disease is in the grain of skin and the texture of the subcutaneous flesh” and guided by the Chinese medicine theory of preventive treatment of diseases combined with the opinions of many classic Chinese medicine experts, we suggested the treatment viewpoint of “treating metabolic diseases from the exterior at the early stage.” We Choose the “Fuling-Zexie decoction,” which is specific to Taiyang-YangMing-Taiyin = combination diseases, as the main prescription for cold dampness, in the formula (see Table 1 [31]): Fuling and Baizhu can dissolve the wetness inside; both Guizhi and Shengjiang work on the exterior to treat the disease in and out simultaneously and pushes the wetness out with Zexie together.

**Table 1:** The ingredients of the Fuling-Zexie decoction

Chinese name	English name	Latin name	Function (in Chinese medicine theory) [31]	Medicinal part	Dosage (g)
Fu ling	Tuckahoe	*Poria cocos (Schw.) Wolf*	Inducing diuresis to drain dampness; strengthening spleen	Dry sclerotia	48
Ze xie	Rhizoma alismatis	*Alisma plantago-aquati ca Linn*	Inducing diuresis to drain dampness; discharge heat	Dry tuber	24
Bai zhu	Largehead Atractylodes	*Atractylodes macrocephala Koidz*	Benefiting vital energy and invigorating the spleen; drying dampness	Tuber root	18
Gui zhi	Cassia twig	*Cinnamomum cassia Presl*	Resolving the flesh and sweating; warming the channels to free the networks vessels; reinforcing yang and activating vital energy	Dry twigs	12
Sheng gancao	Raw Licorice	*Glycyrrhiza uralensis Fisch*	Nourishing spleen and benefiting vital energy; moderating the property of herbs	Dry root	12
Sheng jiang	Fresh ginger	*Zingiber of icinale Rosc*	Resolving the exterior; dissipating dampness	Rhizome	2

## Methods and design

### Study objective

The objectives of the experiment are to study the efficacy and safety data of Chinese medicine therapy for dampness syndrome for hyperuricemia and to screen possible related small molecules/microbial material bases.

### Study design

**Fig. 1:**
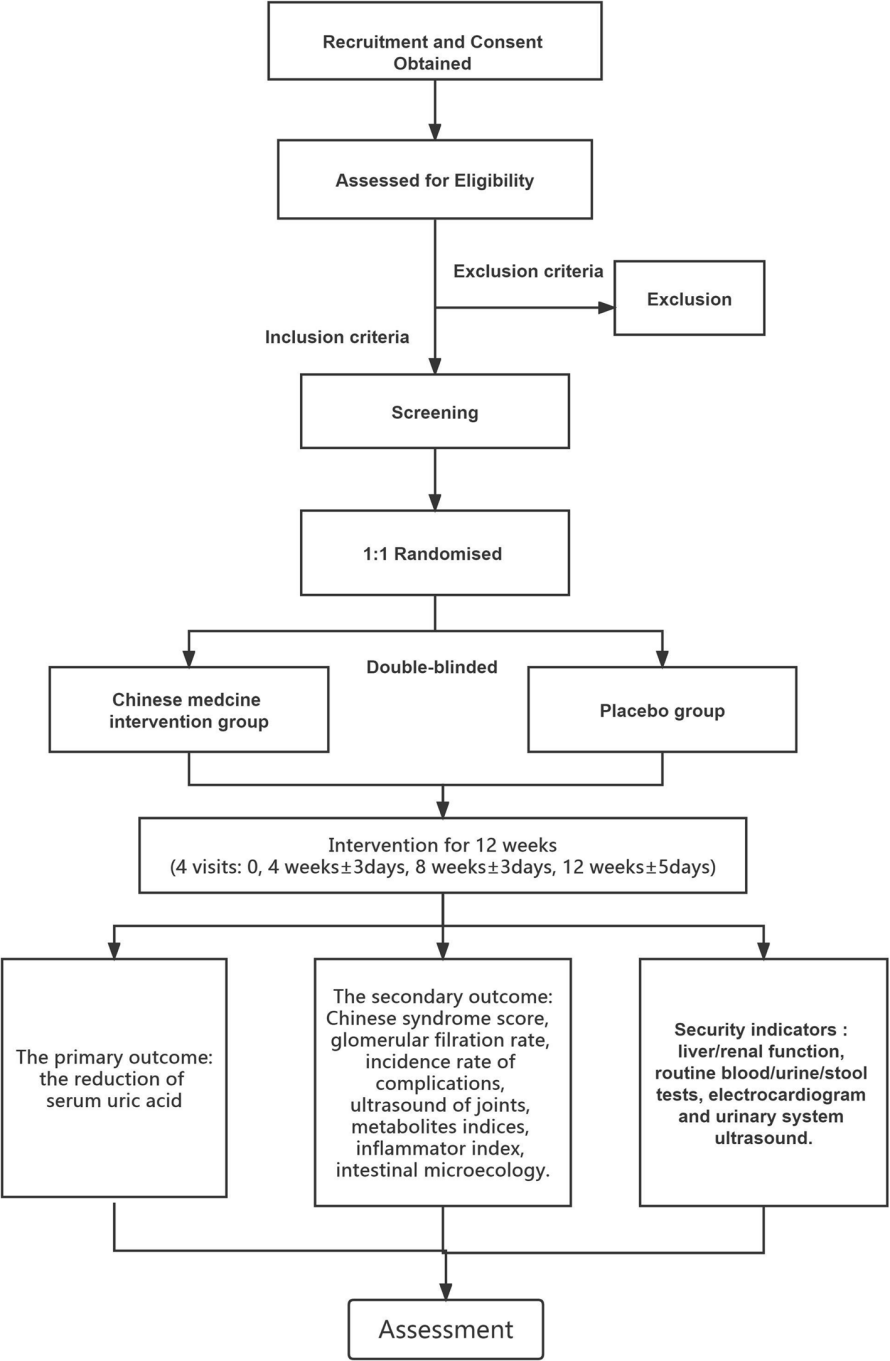
Flow diagram for the study

**Fig. 2:**
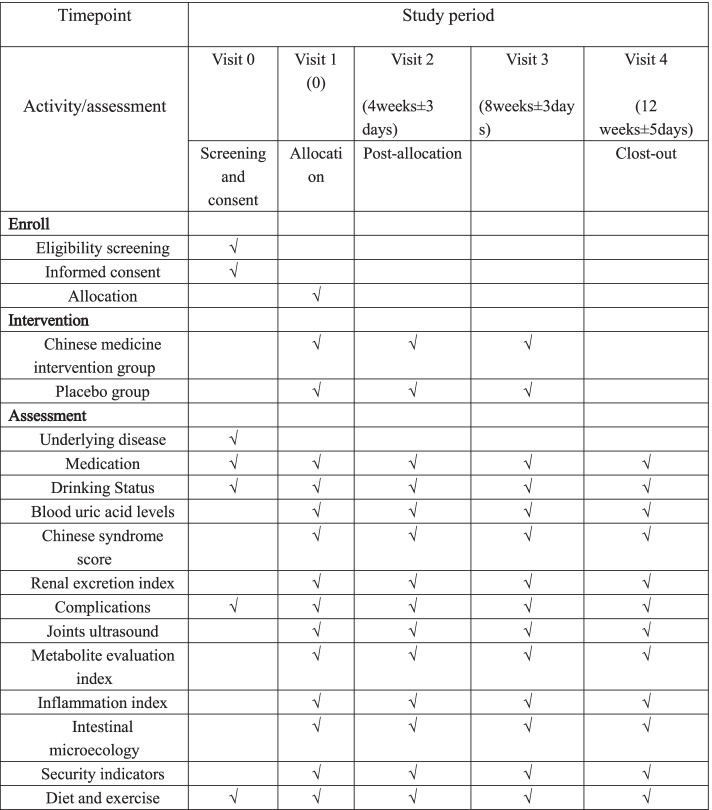
Participant timeline

This is a 12-week, single-center, prospective, double-blinded, randomized, placebo-controlled clinical trial. We used the SPIRIT reporting guidelines to perfect the protocol [32], from March 2020 to December 2021. Thirty individuals with asymptomatic hyperuricemia will be randomly assigned to receive Chinese medicine or a placebo at a 1:1 ratio (Figs. 1 and 2).

### Sample size

This study will enroll 30 subjects. Further large-scale RCT trials will estimate the size of the sample according to the results of the main outcome indicators obtained from this study and use the sample content estimation formula to compare the two means to calculate the sample content, *α* = 0.025, *β* = 0.2. The sample size ratio of the test group and control group is 1. The follow-up rate is set according to the pre-experiment follow-up rate.

### Recruitment

Most asymptomatic patients with hyperuricemia do not seek medical attention, so we will recruit subjects through recruitment advertisements. The advertisements will be placed in the hospital building and be announced through the hospital’s official account.

### Study setting

The Guangdong Provincial Hospital of Chinese Medicine will be the only study setting.

### The study’s key problem

Dampness is involved with the development of hyperuricemia. The key to preventing gout, cardiovascular disease, and urate nephropathy is controlling the serum uric acid level. Can Chinese medicine improve the outcomes of the population with hyperuricemia population and reach the goal of early prevention of disease?

### Eligibility criteria

Inclusion criteria:

1. Aged between 18 and 75 years (including 18 years and 75 years of age);
2. Meet the diagnosis of asymptomatic hyperuricemia;
3. Meet the dampness syndrome inclusion criteria;
4. Sign informed consent.

Exclusion criteria:

1. Secondary hyperuricemia, for instance, secondary to malignancy, hemopathy, nephropathy, and chronic intoxication;
2. History of alcoholism without abstinence in the last 3 months;
3. One or more attacks of gout arthritis in the past;
4. Received urate-lowering drugs or drugs that may increase serum uric acid in the last 4 weeks: (1) antituberculotic drugs, such as pyrazinamide/ethambutanol/isoniazid, (2) low-dose aspirin, (3) loop diuretics, thiazide diuretics and blood pressure medications containing diuretics, (4) niacin, (5) large doses of vitamin C, (6) chemotherapeutic drugs, and (7) cyclosporin;
5. Pregnancy or lactation, or the possibility of conception but failure to use effective contraception;
6. Alanine aminotransferase, aspartate aminotransferase or creatinine levels 2 times or more than the normal value;
7. Serious organic lesions, mental disorders or other reasons a person cannot cooperate with treatment;
8. Allergies or no reaction to the test drug ingredients.

### Randomization and allocation

Adopting simple block group randomization, the allocation ratio will be 1:1. SAS9.2 PROC PLAN will be used to carry out procedure coding and random sequence generation by the methodology team.

The random distribution results will be released through the interactive network response random distribution system for clinical research in Guangdong Provincial Hospital of Chinese Medicine. The random assignment table in the random center will be kept strictly confidential until the end of the study.

After the patients are screened as qualified subjects and have given informed consent, the clinicians apply for the randomization system to obtain the randomization results on the internet. Physicians will execute the results according to random grouping and print and paste them on the corresponding position of the case report form (CRF).

### Blinding

A double-blinded, placebo-controlled design was adopted, and both the investigators and the subjects will be blinded. All data will be evaluated and statistically processed by a third party after clinical research.

The trial will be unblinded if there is a serious adverse event that may be related to the experimental drug, so we can know which group the subject was enrolled in to decide the rescue plan. The trial would be unblinded in the presence of the principal investigators and funders. The time, reason, and procedure of the unblinding and the researchers involved in the unblinding would be recorded. After the trial is unblinded, the subject would be terminated from the study, the experimental data would be included in the safety analysis dataset, and the subject would be given timely treatment and follow-up.

### Comparisons

Many Chinese medicine studies set Chinese medicine plus Western medicine as the experimental group and solely modern medicine as the control group. This may lead to bias. Therefore, we will use a placebo control group for comparison.

### Chinese medicine syndrome subgroups

During the follow-up period, the enrolled subjects will be evaluated to determine whether they should be assigned to a subgroup for dampness-heat syndrome, the definition of which will be drawn up according to “The Guiding Principles for Clinical Research of New Chinese Medicines (trial)” in 2002 edited by Zheng Xiaoyu [33]. In the theory of Chinese medicine, the Fuling-Zexie decoction is no longer suitable when patients have dampness-heat syndrome.

### Interventions

Subjects will be given therapeutic agents (Chinese medicine) or a placebo.

The Chinese medicine intervention group will receive a Fuling-Zexie decoction of one dose a day taken orally after a meal. If the subject entered the subgroup after evaluation, the intervention will be switched to a Huangqin-Shengjiang-Banxia decoction until the combined damp-heat syndrome subsides.

For the placebo group (Chinese medicine placebo), the size, appearance, packaging, and administration method will be the same as those of the therapeutic drugs.

During the trial, the investigators will monitor the dietary and exercise conditions of the subjects every day.

### Elimination and termination criteria

The elimination criteria are as follows: (1) serious violation of exclusion standards after enrollment, (2) not completing the experimental drug administration during the experimental period as required after enrollment, and (3) incomplete one-month follow-up and no test records available for evaluation.

The termination criteria are as follows: (1) a gout attack, (2) severe adverse events, (3) condition deteriorated and required urgent medical attention, (4) the researcher recommended test termination because of the safety of the subjects, and (5) people unwilling to continue to participate in this study for personal reasons.

We will keep in touch with the subjects who drop out to observe any subsequent adverse events and collect data.

### Strategies to improve adherence to interventions

The compliance of investigators and subjects is an essential factor in the clinical research process and clinical effects. To ensure compliance, investigators or the designated representative must give detailed information about the clinical trial to the subjects and obtain informed consent after a sufficient and detailed explanation.

The compliance evaluation of the subjects will mainly be judged from the status recorded in the subject’s log card and the questions asked by the researchers.

### Plans to promote participant retention and complete follow-up

First, the investigators will keep in touch with the subjects, and regular telephone follow-ups and reminders for follow-up visits will be implemented. Second, our research system will issue the prescription a day in advance. Third, the subjects will receive free tests. Fourth, transportation subsidies will be given after each follow-up visit to encourage subjects to remain engaged and complete follow-up surveys.

### Outcome measures

#### Primary outcome measure

The reduction of serum uric acid is the primary outcome measure.

#### Secondary outcome measures

The secondary outcome measures are as follows: (1) Chinese syndrome score, measurement of the channels and collaterals, and tongue diagnosis evaluation; (2) glomerular filtration rate, urinary β2 microglobulin, and uric acid excretion fraction; (3) incidence rate of complications, such as gout, hyperlipidemia, cardiovascular events, diabetes, etc.; (4) ultrasound of both ankles and the first metatarsophalangeal joints [34]; (5) evaluation index of metabolites, including blood and urine metabolites; (6) inflammatory index: hsCRP, TNF-a, IL-6, and IL-8; and (7) intestinal microecology (note: this index is tested by a separate participant).

### Security indicators

Security indicators include the following: (1) liver function (alanine aminotransferase ALT, aspartate aminotransferase AST) and renal function (blood urea nitrogen, creatinine); (2) routine blood tests, routine urine tests, routine stool tests + occult blood; and (3) an electrocardiogram and ultrasound of the urinary system.

### Data collection and analysis

Within the 12-week study period, there will be 4 visits: the first at week 0, the second at 4 weeks ± 3 days, the third at 8 weeks ± 3 days, and the fourth at 12 weeks ± 5 days. The data collection and completion of the CRF will be performed by two research assistants who will be blinded to the grouping of the participants. The EPIdata3.1 database software package will be used to establish a database for data entry, and double input data will be used for data entry.

The clinical trial monitors will check the quality of data entry regularly. The database after verification will then be checked by logic inspection, and the outliers will be checked with the original laboratory documents, and the final checked database will be converted into an DATABASE in the format of the SPSS statistical software package and locked for statistical analysis.

### Statistical analysis

All statistical tests will be two-sided, and *α* = 0.05 will be considered statistically significant.

We will use a *t*-test (or Wilcoxon matched-pairs signed-ranks test if the data are non-normally distributed with uneven variances) to compare the decrease in uric acid between two groups.

For secondary outcomes, the *t*-test and Wilcoxon test will be conducted to compare the differences in measurement data (such as index mean, standard deviation, and median) between the groups. Repeated measures analysis of variance will be used to compare multiple measures of the scale and symptom rating among groups. The chi-square test (or Fisher’s exact test) and 3×C table will be utilized to determine the differences in the proportion, frequency, and total effective rate of the indices in the Chinese medicine group and placebo group.

To analyze the influencing factors of curative effects, non-conditional logistic regression analysis will be performed. For missing data from long-term follow-up, the last carry-over method (LOCF) will be used.

### Safety analysis

Safety data consist of adverse events and clinical laboratory tests. We will describe specific manifestations of adverse reactions and compare the incidence of adverse events and severe adverse events between the two groups. Changes in laboratory test results before and after the test, abnormal changes, and their relationship with Chinese medicine will be analyzed.

### Compliance analysis

The compliance among the subjects included in the study will be analyzed by descriptive analysis, while an intergroup descriptive analysis will be performed on the baseline information of the drop-out cases in both groups.

### Adverse events management

The type, degree, occurrence time, duration, treatment measures, treatment process, and follow-up results of adverse events that occurred during the trial will be recorded in the case report form, and the correlation between the adverse events and the experimental drugs will be evaluated on the basis of comprehensive consideration of the complications and combined use of drugs, which will also be recorded in detail by the physician.

For non-serious adverse events, the observation physician may decide whether to suspend observation based on the disease condition. Cases in which drugs are withdrawn due to adverse events will be followed up and the lab security indicators will be reexamined.

For serious adverse events, drug use will be stopped immediately. Regardless of whether it is related to the experimental drug, the investigators will report to the Ethics Committee of Guangdong Province Hospital of Chinese Medicine within 24 h after its occurrence.

If a serious adverse event occurs during the course of the study, the research team and the hospital will be responsible for subsequent treatment and financial compensation.

### Protocol amendments

If a protocol change is required, the risk to the subject will be fully assessed and reviewed by the Ethics Committee, and all members will be notified after the new scheme has been approved through email and face-to-face meetings.

### Confidentiality

Subjects have independent codes and only enter codes when uploading test data. An encryption system will be utilized to upload and store data. The final statistical process will be administered by a third party.

## Discussion

The guidelines in different countries vary regarding asymptomatic hyperuricemia, which can lead to gout and other diseases. Social and ethnic backgrounds as well as the different development of medical ideas may prone to these differences [6]. The lack of double-blinded randomized controlled trials is another major factor. Additionally, the benefit-risk ratio of pharmacological treatments (such as febuxostat and allopurinol) is an issue that clinicians should consider. Thus, the aim of our research is not only to determine the effect and mechanism of Chinese medicine in the treatment of asymptomatic hyperuricemia but also to provide more evidence-based research for clinical work.

Based on previous years of clinical experience and cross-sectional investigation and analysis, the motive for this study is to carry out pre-research to proceed with a study aimed at people with hyperuricemia and dampness syndrome in Gouangdong; on the basis of the pre-research, the sample size will be expanded and the randomized controlled double-simulation clinical trial will be conducted to obtain high-level evidence-based research of Chinese medicine in the treatment of hyperuricemia and dampness syndrome and thus form an effective early Chinese medicine intervention plan for the population with hyperuricemia and dampness syndrome. Through this pilot study and further large-scale research, we want to provide more effective and low-risk treatment options to benefit patients. We hope to determine whether controlling the level of serum uric acid by using Chinese medicine can prevent or delay gout and other complications to reduce the pain and medical costs of patients.

As a pilot study, the limitations are obvious. There will be bias due to the small number and single data source. As the first double-blinded, randomized, placebo-controlled clinical trial to study the treatment of asymptomatic hyperuricemia with Chinese medicine, we are still not sure whether the follow-up period is long enough to see the decoction take effect. There are more questions that need to be studied, such as the level of serum uric acid needed for Chinese medicine intervention, when to withdraw the decoction, and whether the benefits of treatment with Chinese medicine can outweigh the potential medical costs in the long term. We assume that in the future, there will be more formulae and decoctions proven effective or explored.

## Trial status

This trial information is available on chictr.org.cn, protocol version 1.4, November 30, 2020. The study was funded on February 25, 2020. Our trial enrollment period was from March to August 2021.

## Supplementary Information

**Additional file 1: Appendix 1.** Model-informed consent form.

**Additional file 2: Appendix 2.** SPIRIT checklist.

## Data Availability

The data will not be shared because this is a preliminary experiment. The research unit will have access to the final trial dataset.
