# Supplementary material for: Evaluation of the safety and efficacy of a Fuling-Zexie decoction for people with asymptomatic hyperuricemia: protocol for a prospective, double-blinded, randomized, placebo-controlled clinical trial
Source: Trials. 2022 Jun 20;23:517. doi: 10.1186/s13063-022-06479-3 (PMC9208148; doi:10.1186/s13063-022-06479-3)
Supplement: Supplementary file 1 — Additional file 1: Appendix 1. Model-informed consent form. [file 13063_2022_6479_MOESM1_ESM.docx]

知情同意书

**尊敬的患者：**

您的医生已经确诊您患有高尿酸血症。

我们将邀请您参加一项无症状高尿酸血症早期干预研究，本研究将观察中药祛湿方及其模拟剂对于湿证高尿酸血症患者的临床疗效及安全性。该课题是“中医湿证省部共建实验室”研究中的无症状高尿酸血症研究项目。

在您决定是否参加湿证高尿酸血症早期与干预临床试验前，请详细阅读下述内容，将有助您了解该项研究的程序和内容，参加该研究可能带来的益处，可能对您带来的不便等。如果您愿意，也可以和亲属、朋友一起讨论，或者请医生予以解释，以帮助您做出决定。

**项目介绍**

一、目的与意义

目前中国高尿酸血症呈现高流行、年轻化的趋势，高尿酸血症是痛风性关节炎发病的生化基础，也是多种心血管危险因素及相关疾病（代谢综合征、2型糖尿病、高血压、心血管事件及死亡、慢性肾病等）的独立危险因素。但对于无症状的早期高尿酸血症的治疗，因药物使用的不良反应，目前并不推荐积极使用降尿酸药物。中医认为高尿酸血症为湿浊之邪所致，本课题拟采用随机双盲安慰剂对照的临床研究方法，纳入30例湿证高尿酸血症人群，给予中药复方茯苓泽泻汤剂／黄芩加生姜半夏汤及茯苓泽泻汤／黄芩加生姜半夏汤模拟剂口服治疗，观察中药治疗湿证高尿酸血症的初步疗效和安全性数据，为大样本量的临床随机对照试验提供临床基础及样本量估算依据。

本研究将在广东省中医院开展，计划招收30名受试者自愿参加。广东省中医院风湿专科创立于1986年，经过数十年的发展，形成以3名主任医师、4名副主任医师，4名主治医师，6名住院医师的技术人才梯队，近年来承担有关风湿类疾病的各级科研课题30余项，其中国家级课题3项，荣获医疗成果5项，科技进步奖二等奖1项，在国际国内学术期刊发表论文百余篇，主编及参编风湿病专科论著10余部，为广东省中医药管理局重点学科建设单位。

本研究的主要负责人是何晓红副主任医师，该研究者是广东省中医院风湿科副主任中医师，从事风湿病的临床科研工作十余年，具有丰富的临床诊疗与研究经验。

本项研究已经得到广东省中医院伦理委员会批准。广东省中医院伦理委员会已经审议此项研究是遵从赫尔辛基宣言原则，符合医学伦理的要求。

1. 哪些适合参加本研究

1.如果您符合以下全部条件可以参加本研究：

①年龄为18岁-75周岁（含18岁和75岁）者；

②符合无症状高尿酸血症诊断者；

③符合湿证入组标准者；

④签署知情同意书者。

2. 但如果您同时存在以下任意一种情况则不宜参加本研究，因为合并这些情况参加研究可能给您带来额外的风险：

①继发性高尿酸血症人群，如继发于恶性肿瘤、血液病、肾脏疾病、慢性中毒的高尿酸血症等；

②有酗酒史且近3个月未戒酒者；

③既往有痛风性关节炎发作1次或1次以上者；

④受试者近4周有服用降尿酸药物，或可引起血尿酸增高的药物：（1）抗结核药：吡嗪酰胺/乙胺丁醇/异烟肼，（2）小剂量阿司匹林，（4）袢利尿剂和噻嗪类利尿剂以及含有利尿剂成分的降压药，（5）烟酸，（6）大剂量维生素C，（7）肿瘤化疗药，（8）环孢素；

⑤妊娠期及哺乳期，或有受孕可能而未能采取有效避孕措施者；

⑥谷丙转氨酶或谷草转氨酶或肌酐高于正常值2倍或2倍以上者；

⑦合并严重器质性病变、精神意识障碍或其他原因无法配合治疗的受试者；

⑧对试验药物成分过敏或无效者。

三、如果参加本项目将需要做什么？

1.在您入选研究前，您将接受以下检查以确定您是否可以参加研究：

医生将询问、记录您的病史，判断您的中医证型，对您进行相应体格检查。您需要进行血常规、大便常规、尿常规、肝功能、肾功能、血尿酸、血糖、血脂、超敏C反应蛋白、心电图检查、泌尿系彩超及关节超声。

2.若您以上检查合格，将按以下步骤进行研究

研究开始将根据计算机提供的随机数字，决定您中药复方茯苓泽泻汤剂／黄芩加生姜半夏汤或茯苓泽泻汤／黄芩加生姜半夏汤模拟剂治疗，参加这项研究的患者分别有50%的可能性被分入治疗组和对照组，您和您的医生都无法事先知道和选择任何一种治疗方法，治疗观察将持续12周。

治疗开始前：入选后您还需要配合完善24小时尿检查24小时尿尿酸，经络测评、舌诊测评，湿证量表填写、网络上传舌像图片。

治疗后第4周：您应到医院就诊，并如实向医生反映病情变化，医生将收集您的病史及并要求您完善血常规、大便常规、尿常规、肝功能、肾功能、血尿酸、血糖、血脂、超敏C反应蛋白、IL-6、IL-8、TNF-a等检查。

治疗后第12周：这时候研究结束了。您应该到医院就诊，医生将询问记录您病情的变化，给您做体格检查，还将做血常规、大便常规、尿常规、肝功能、肾功能、血尿酸、血糖、血脂、超敏C反应蛋白、IL-6、IL-8、TNF-a、心电图检查、泌尿系彩超及关节超声、24小时尿酸、络测评、舌诊测评，湿证量表填写、网络上传舌像图片。

3.需要您配合的其他事项

您需要按医生和您的约定的时间来医院就诊。您的随访非常重要，因为医生将判断您接受的治疗是否真正起作用。

您需要按医生指导用药，您在每次随访时都必须归还未用完的药物及其包装，并将正在服用的其他药物带来，包括您有其他合并疾病须继续服用的药物。

在研究期间您不能使用治疗高尿酸血症的其他药物。如您需要进行其他治疗，请事先与您的医生取得联系。

4.您参加试验可能被终止的预期情况和/或原因

（1）出现严重不良事件；

（2）依从性差（受试者在药物的使用、接受访视、随访等方面不能依从临床试验方案执行），多次沟通、电话随访无效者，予以病例终止；

（3）受试者在试验期间病情急剧恶化，并且不能排除其病情变化与试验无关，则终止此临床研究，并加强治疗方案；

（4）患者撤回知情同意书，则病例观察终止；

（5）在研究中发现所定临床研究方案有重大失误，难以评价药物疗效；或对一项设计较好的方案，在实施中发生了重大偏差，难以评价药物效应。如果发生了上述情况，研究者有权不征得您的同意而终止您参加本项研究。

四、为该研究捐献的样本和提供的信息将如何处理？

得到您的样品后，我们的团队会进行前期处理（包括对您血液进行离心、分装），再进行相关储存分析，以科学的处理方式，最终实现研究的目的，完成论文的编写。实验中所需的生物样本留取及保存管理按照广东省中医院生物样本库管理SOP执行。

五、参加研究可能的受益

您和社会将可能从本研究中受益。此种受益包括您的病情有可能获得改善；本项研究还有助于确定哪种治疗方法可以更安全有效地治疗与您患有相似病情的其他受试者。

六、参加研究可能的不良反应、风险和不适、不方便

所有治疗药物都有可能产生副作用。如果在研究中您出现任何不适，或病情发生新的变化，或任何意外情况，不管是否与药物有关，均应及时通知您的医生，他/她将对此作出判断和医疗处理及判断您是否适合继续参加研究。

您在研究期间需要按时到医院随访，做一些体格检查和实验室检查，这些都可能给您造成麻烦或带来不方便。

此外，任何治疗都可能出现无效的情况，以及因治疗无效或者因合并其他疾病等原因而导致病情继续发展。这是每个就医患者都将面临的治疗风险，即使不参加本项临床研究，治疗风险都将存在。在研究期间，如果医生发现本项研究所采取的治疗措施无效，将会中止研究，改用其他可能有效的治疗措施。

七、有关费用

本研究课题组将支付您参加本项研究期间所做的与研究有关的检查（血常规、尿常规、便常规、肝功能、肾功能、血脂、血糖、血尿酸、C反应蛋白、心电图、24小时尿尿酸、关节超声、泌尿系彩超）费用，并免费提供治疗药物。

如果发生与试验相关的损害，课题组将支付您的医疗费用以及按照法律法规规定给予相应的经济补偿。

如果您同时合并其他疾病所需的治疗和检查，将不在免费的范围之内。

如果因治疗无效，我们将提供中西药结合方案等其他治疗手段，但非免费治疗。

八、个人信息保密的吗？

任何有关本项研究结果的公开报告将不会披露您的个人身份。即在适用法律和（或）法规准许的范围内，受试者的身份仍然是保密的。我们将在法律允许的范围内，尽一切努力保护您个人医疗资料的隐私。

除本研究外，有可能在今后的其他研究中会再次利用您的医疗记录。您现在也可以声明拒绝除本研究外的其他研究利用您的医疗记录和标本。

九、怎样获得更多的信息？

您可以在任何时间提出有关本项研究的任何问题。您的医生或研究者将给您留下他/她的电话号码以便能回答您的问题。

如果您对参加研究有任何抱怨，请联系广东省中医院伦理委员会办公室（联系电话：020-81887233-35943）。

如果在研究过程中有任何重要的新信息，可能影响您继续参加研究的意愿时，您的医生会及时通知您。

十、可以自愿选择参加研究和中途退出研究

是否参加研究完全取决于您的自愿。您可以拒绝参加此项研究，或在研究过程中任何时间退出本研究，这都不会影响您和医生间的关系，也不会影响对您的医疗或有其他方面利益的损失。

如果您需要其他诊断/治疗，或您没有遵守试验计划，或者有任何其他合理原因，您的医生或者研究者可以终止您继续参加试验。

如果您因为任何原因从研究中退出，您可能被询问有关您使用试验药物的情况。如果医生认为需要，您也可能被要求进行实验室检查和体格检查。这对保护您的健康有利。

十一、现在该做什么？

在您做出参加研究的决定前，请尽可能向您的医生询问有关问题，直至您对本项研究完全理解。

是否参加本项研究由您自己决定。您可以和您的家人或者朋友讨论后再做出决定。

感谢您阅读以上材料。如果您决定参加本项研究，请告诉您的医生或研究助理，他她会为您安排一切有关研究的事务。

请您保留这份资料。

知情同意书·同意签字页

Signature Leaflet for Informed Consent

**临床研究项目名称：**

**申办者/课题下达单位：广东省中医院**

**伦理审查批件号：**

同意声明

我已经阅读了上述有关本研究的介绍，而且有机会就此项研究与医生讨论并提出问题。我提出的所有问题都得到了满意的答复。

我知道参加本研究可能产生的风险和受益。我知晓参加研究是自愿的，我确认已有充足时间对此进行考虑，而且明白：

● 我随时可以向医生咨询更多的信息。

● 我可以随时退出本研究，而且不会受到歧视或报复，医疗待遇与权益不会受到影响。

我同样清楚，如果我中途退出本研究，特别是由于药物的原因使我退出研究时，我若将病情变化告诉医生，完成相应的体格检查和理化检查，这将对我本人和整个研究十分有利。

如果因患病我需要采取任何其他的药物治疗，我会在事先征求医生的意见，或在事后如实告诉医生。

我同意药品监督管理部门、伦理委员会或申办者代表查阅我的研究资料。

我将获得一份经过签名并注明日期的知情同意书副本。

最后，我决定同意参加本项研究。

患者/受试者 签名： 年 月 日

监护人/授权委托人（如适用） 签名： 年 月 日

联系电话： 手机号：

我确认已向患者解释了本试验的详细情况，包括其权利以及可能的受益和风险，并给其一份签署过的知情同意书副本。

医生签名： 日期： 年 月 日

医生的工作电话： 手机号：

广东省中医院伦理委员会办公室联系电话：020-81887233-35943
